# Elevated Corticosterone Levels and Changes in Amphibian Behavior Are Associated with *Batrachochytrium dendrobatidis* (*Bd*) Infection and *Bd* Lineage

**DOI:** 10.1371/journal.pone.0122685

**Published:** 2015-04-20

**Authors:** Caitlin R. Gabor, Matthew C. Fisher, Jaime Bosch

**Affiliations:** 1 Department of Biology, Texas State University, San Marcos, Texas, United States of America; 2 Department of Infectious Disease Epidemiology, Imperial College London, Norfolk Place, London, United Kingdom; 3 Museo Nacional de Ciencias Naturales, CSIC, c/ José Gutiérrez Abascal 2, Madrid, Spain; Smithsonian's National Zoological Park, UNITED STATES

## Abstract

Few studies have examined the role hormones play in mediating clinical changes associated with infection by the parasite *Batrachochytrium dendrobatidis* (*Bd*). Glucocorticoid (GC) hormones such as corticosteroids (CORT) regulate homeostasis and likely play a key role in response to infection in amphibians. We explore the relationship between CORT release rates and *Bd* infection in tadpoles of the common midwife toad, *Alytes obstetricians*, using a non-invasive water-borne hormone collection method across seven populations. We further examined whether tadpoles of *A*. *muletensis* infected with a hypervirulent lineage of *Bd*, *Bd*GPL, had greater CORT release rates than those infected with a hypovirulent lineage, *Bd*CAPE. Finally, we examined the relationship between righting reflex and CORT release rates in infected metamorphic toads of *A*. *obstetricans*. We found an interaction between elevation and *Bd* infection status confirming that altitude is associated with the overall severity of infection. In tandem, increasing elevation was associated with increasing CORT release rates. Tadpoles infected with the hypervirulent *Bd*GPL had significantly higher CORT release rates than tadpoles infected with *Bd*CAPE showing that more aggressive infections lead to increased CORT release rates. Infected metamorphs with higher CORT levels had an impaired righting reflex, our defined experimental endpoint. These results provide evidence that CORT is associated with an amphibian’s vulnerability to *Bd* infection, and that CORT is also affected by the aggressiveness of infection by *Bd*. Together these results indicate that CORT is a viable biomarker of amphibian stress.

## Introduction

The disease chytridiomycosis is caused by the chytrid fungus *Batrachochytrium dendrobatidis* (*Bd*), a parasite that has contributed to the global decline in amphibian populations worldwide [1–3]. The *Bd* pathogen invades the skin, and in doing so disrupts electrolyte transport, ultimately leading to cardiac failure and death [4]. The disease also suppresses appetite, disrupts righting reflex and affects skin shedding [4]. The role that hormones play in mediating these clinical changes has not been fully examined; however, glucocorticoid (GC) hormones appear to link different aspects of infection owing to their key role in regulating homeostasis. Elevated corticosterone (CORT), the main amphibian GC, is associated with infection levels and disease status for *Bd* [5–7]. Chronic elevation of CORT can influence development, growth, metabolism, appetite, and immunity and these factors can lead to increased susceptibility to disease [8]. Because chronic elevation of CORT can inhibit immune responses, CORT likely influences host-pathogen interactions via changes in the physiology of animals in natural populations [9].

Elevated CORT levels can be driven by a myriad of factors. Anthropogenic factors such as invasive species, habitat destruction, environmental contamination, and changes in the climate and atmosphere are largely associated with changes in baseline CORT levels [9]. Environmental stressors can impair defenses produced by the host against *Bd* [10, 11] and this can lead to higher infection burden. Moreover, elevated CORT can accelerate metamorphosis and comes at the cost of additional immune responses [12, 13]. In sum, numerous factors may be associated with elevated CORT all of which may drive levels of *Bd* infection.

There is both species and population-level variation in susceptibility to infection with *Bd* [14]. For example, some populations of the common midwife toad (*Alytes obstetricans*) show high rates of mortality and population crashes owing to infection by *Bd*, whereas other infected populations appear stable [15–17]. Environmental variables such as precipitation, temperature and elevation can affect host susceptibility [18, 19]. Elevation influences *Bd* dynamics across a range of ecosystems [20–25], but not all [26]. CORT increases with increasing elevation in one amphibian species [20], but CORT was negatively related to elevation in many other amphibians reviewed by [27] possibly due to greater temperature variability at higher elevation (or lower). A further complexity in the relationship between elevation and CORT is that *Bd* is composed of multiple divergent lineages [28, 29] and some of these lineages vary in their virulence thus affecting susceptibility. Farrer et al. [28] showed that a lineage emerging worldwide, *Bd*GPL, is hypervirulent compared to *Bd*CAPE; both these lineages infect the amphibian species that we study. While a link between changes in CORT and response to infection by *Bd* has been suggested, it is not clear how CORT, *Bd* infection status and *Bd* lineage interact.

CORT levels in populations of common midwife tadpoles, *A*. *obstetricans* are higher in *Bd* infected populations than in non-infected populations, as well as in one *Bd* infected population of *Alytes muletensis*, the Mallorcan midwife toad, compared to an uninfected population of that species [5]. The mean CORT release rates in the infected population of *A*. *muletensis* were significantly lower than the infected population of *A*. *obstetricans*, and we proposed that these differences were due to the lineage of *Bd* infecting the population. Specifically, *A*. *obstetricans* is infected with the hypervirulent lineage, *Bd*GPL, whereas the populations of *A*. *muletensis* are infected with an African-type lineage, *Bd*CAPE. Tadpoles of *A*. *muletensis* exposed to *Bd*GPL have significantly greater mortality than those exposed to *Bd*CAPE, directly demonstrating this hypervirulent phenotype is manifest in *A*. *muletensis* [30].

In this study, we test the hypotheses that CORT affects vulnerability to *Bd* infection and that *Bd* infection affects CORT release rates. First, we examined if the association between *Bd* infection and greater CORT levels is found across seven additional populations of *A*. *obstetricans*. Second, we exposed tadpoles of *A*. *muletensis* to *Bd*GPL and *Bd*CAPE lineages and examined CORT release rates. Finally, to further examine the physiological effects of higher CORT we also examined the righting behavior of *Bd* infected metamorphs of *A*. *obstetricans* to determine whether there were measurable behavioral effects *in situ*. This study provides a better understanding of the mechanistic factors associated with the dynamics of *Bd* infections.

## Materials and Methods

### Experiment 1: Relationship between *Bd* and CORT across populations of *Alytes obstetricans*

**Fig 1:**
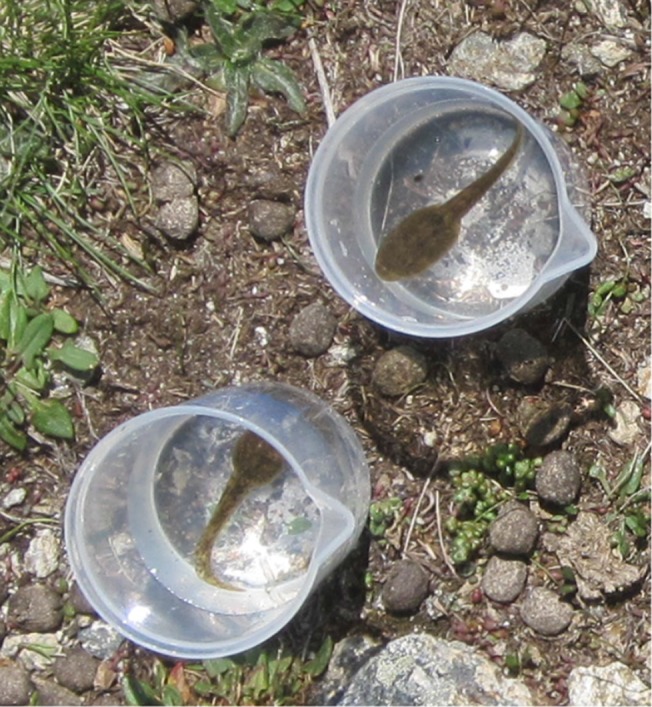
Tadpoles of *Alytes obstetricans* in beakers during water-borne hormone collection.

We sampled *Alytes obstetricans* (Fig 1) from seven field sites from 6 June 2012–24 July 2012. We sampled *A*. *obstetricans* tadpoles at three *Bd*GPL infected populations that have been infected at high intensity for many years (Arlet, French Pyrenees: 42.838591, -0.61466, 1986 m absl; Ansabere, French Pyrenees: 42.887757, -0.708339, 1859m absl; Acherito, Spanish Pyrenees: 42.879258, -0.708811, 1875m absl). We also sampled *A*. *obstetricans* tadpoles at two populations that are consistently infected with *Bd*GPL every year during colder months (Bosch unpub. data; [25]) but showed no active infection when we tested them during the warmer summer months (La Huelga, Picos de Europa National Park:43.265590, -4.952379, 1056m absl; Toro, Zamora: 41.377163, -5.452001, 751m absl).We also sampled from two populations that have never been infected with *Bd* (Igüedri, Picos de Europa National Park: 43.146026, -4.773773, 1283m; Ibonciechu, Spanish Pyrenees: 42.800817,-0.310879, 2.230m absl; Walker *et al*., 2010). We used a dip net to collect tadpoles from each population (*n* = 15–20 for each population) and then placed them in 100ml beakers (cleaned in advance with 95% ethanol) filled with 40 ml of spring water for 1 h to collect water-borne hormones. In most of the populations, before releasing tadpoles back into their pond, we swabbed the keratinized mouthparts to determine *Bd* load, measured the snout-vent length (SVL), and recorded their approximate Gosner stage [31]. We undertook all manipulations wearing gloves. We do not have direct data on the infection levels of the individuals collected at Ansabere and Acherito but we confirmed that they were *Bd* infected in August 2012 with a prevalence of 100%. We sampled tadpole hormones between 1100 -1530h to minimize effects of circadian variation in CORT. We placed samples in coolers with ice for transport back to the laboratory.

### Experiment 2: CORT response to *Bd* lineages in *Alytes muletensis*

To examine the CORT response to different lineages of *Bd* we exposed captive bred *A*. *muletensis* tadpoles (Gosner stage 26–30) to one of three randomly assigned treatments: (1) *Bd*GPL, UK TvB isolate (*n* = 17), (2) *Bd*CAPE, TF5a1 isolate (*n* = 19), and (3) sham exposure (filtered media that had *Bd* grown and filtered; *n* = 17). The experiment was run from 19 March—4 May 2012. We individually housed tadpoles in 0.7-L plastic containers then exposed them to 0.5 ml of 12,000 zoospores four separate times on 27 March, 30 March, 3 April and 6 April. We obtained hormone data on 10 April 2012 by placing each tadpole from the three treatments in a clean 100 ml beaker with 40 ml of aged tap water for 1h. A few water-borne hormone samples were lost due to spilling. After obtaining water-borne hormones we obtained the animal’s final mass (g) and recorded their Gosner stage [31]. To determine infection status we then swabbed the mouthparts of tadpoles to determine *Bd* load on 13 April 2012 (time 1) and again on 4 May 2012 (time 2) using sterile rayon-tipped swabs (MW100; Medical Wire & Equipment Co, Corsham, UK). At the end of the experiment animals were euthanized in MS222 under a UK Home Office licence.

### Experiment 3: Righting reflex of metamorphic *Alytes obstetricans*

To examine the physiological effects of CORT on *Bd* infected metamorphic *A*. *obstetricans* we returned to a *Bd* infected population in the French Pyrenees, Arlet on 17 August 2012. We searched for metamorphs and placed each one (*n* = 20) in 20 ml of spring water in a clean 100 ml beaker for 1 h. After obtaining water-borne CORT samples, we turned each metamorph over onto its back and observed whether it could right itself (correct its body position). Healthy metamorphs do this with no problem [4]; however, the inability of an animal to right itself after three inversions is considered to be an indication of lethal chytridiomycosis and constitutes an experimental end point. We continued to flip the metamorph on its back each time it righted itself for 1 min. We recorded the total number of times the metamorphs were able to correct their body position per min. We then obtained SVL and swab samples wearing disposable gloves by firmly running the same sterile rayon-tipped swabs over the lower abdomen, drink patch, all four limbs and digits of all limbs to determine *Bd* loads by qPCR. No animals were euthanized and all animals were returned to the spot where they were found.

### Quantitative PCR method

We screened for *Bd* infection using a quantitative real-time polymerase chain reaction (qPCR) protocol [32]. We extracted nucleic acids from samples following [32], and diluted the extractions 1:10 before qPCR amplification. Samples were examined in duplicate with *Bd* genomic equivalent (GE) standards of 100, 10, 1, and 0.1 GE. Samples were considered negative if a follow-up third amplification did not result in an amplification profile.

### Ethics Statement

This study was carried out with approval from Texas State University Institutional Animal Care and Use Committee (approval number 0620_0720_19) and by the animal welfare Consejerias de Medio Ambiente of Aragón (permit LCR/mp 24/2012/645), Castilla y León (permit IS/pa EP/CyL/79/2012), Picos de Europa National Park (permit CO/09/032/2012) and Les Pyrénéss National Park (permit 2012/70).

### Hormone extractions and validation

We stored water samples at -20°C until they were thawed for extraction in Spain. We extracted hormones from water using C 18 solid phase extraction (SPE) columns (Waters Inc.). After extracting into the column, we stored the SPE columns at -20°C until we were ready for methanol elution at Texas State University. All methods follow from [5]. We measured CORT release rates using enzyme-immunoassay (EIA) kit (Cayman Chemicals Inc.) in duplicate with a 96 well plate reader set at 415 nm (BioTek Powerwave XS). We did not subtract the CORT level measured in the water because we used the same water for all populations in Experiment 1.

We previously validated the use of water-borne hormones on EIA plates and the positive correlation between plasma CORT and water-borne CORT release rates for *A*. *obstetricans* [33]. Gabor et al. [5] additionally validated the use of the CORT EIA kits with water-borne hormones for *A*. *muletensis*.

### Statistical analyses

We normalized the CORT data in experiment 1 and 3 by dividing by the SVL of the tadpoles. In experiment 2, only mass was obtained so we divided the CORT data by mass. Because Gabor et al. [5] used data normalized with SVL we used the same again here. Results do not change whether we use SVL or mass in experiments 1 and 3. In both species of tadpoles there is a strong positive relationship between SVL and mass (Linear regression: *A*. *obstetricans*: r^2^ = 0.84, *n* = 109, *P* < 0.0001, this data, and as [5] found for *A*. *muletensis*). CORT values were not normally distributed in any experiment so we LN transformed the normalized CORT data to meet the assumptions of parametric analyses in all experiments. In experiment 1, we examined the relationship between elevation (m) and the mean CORT value for each population because CORT is predicted to change with elevation. We found a significantly positive relationship between elevation and CORT (Kendall’s tau = 0.45, *n* = 111, *P*< 0.0001). Given this relationship, we used an ANCOVA to examine the relationship between CORT release rates as the dependent variable and infection status (infected or uninfected) as the independent variable with elevation as a covariate. To examine the relationship between *Bd* load (GE value) and CORT values we used Kendall’s tau because GE did not meet the assumptions of parametric statistics even when transformed. We used a logistic regression to examine the relationship between elevation and infection status when controlling for CORT. In experiment 2, we used an ANOVA to examine the relationship between CORT values and *Bd* lineage treatment followed by Tukey’s HSD. In experiment, 3 we used a pairwise correlation to examine the relationship between flips/min and CORT and Kendall’s tau between *Bd* load and CORT. We used two-tailed *P* values and alpha was set at 0.05.

## Results

### Experiment 1: Relationship between *Bd* and CORT across populations of *Alytes obstetricans*

**Fig 2:**
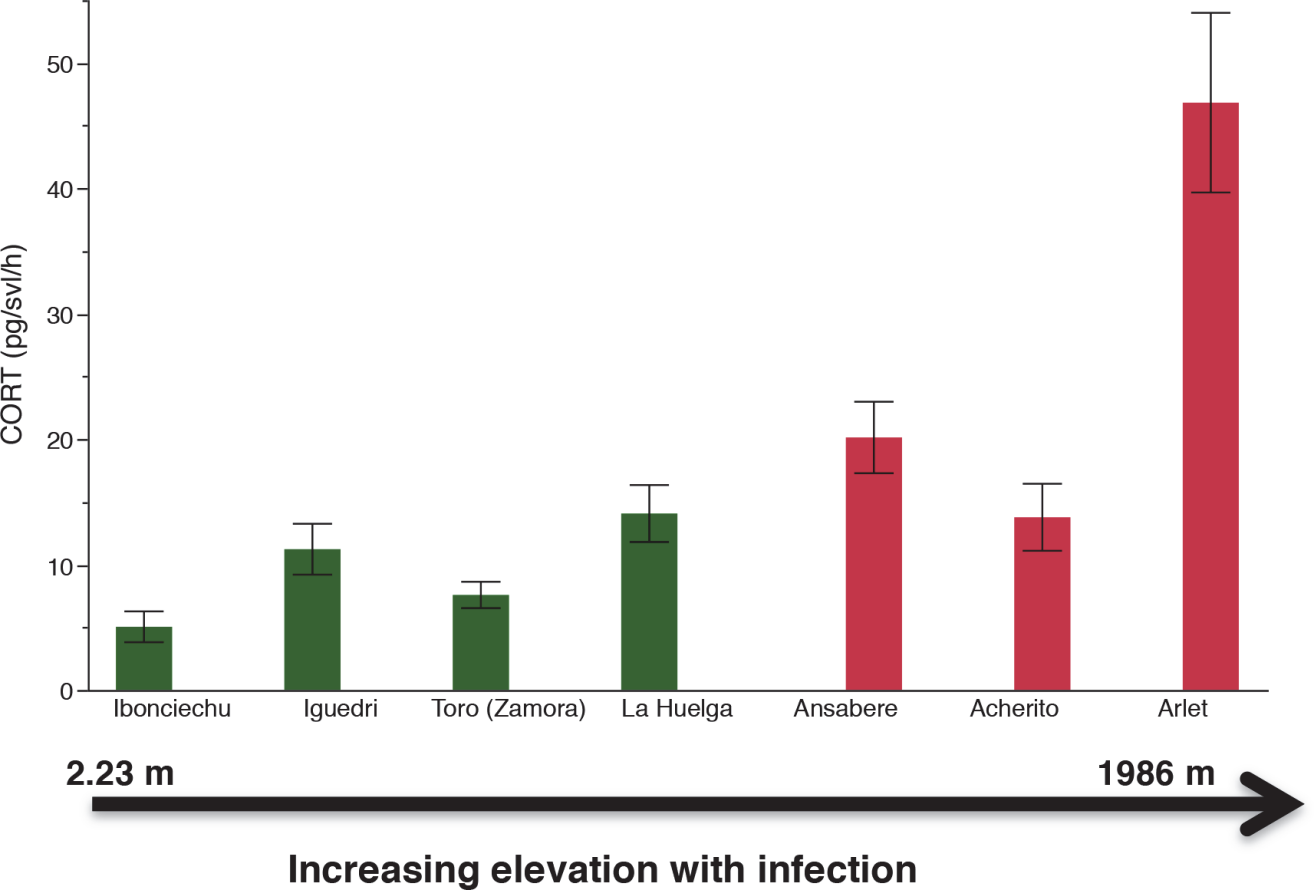
Mean (± SE) water-borne CORT release rates of tadpoles with infection and elevation. Red bars indicate infected populations and green bars indicate uninfected populations of *Alytes obstetricans*.

**Fig 3:**
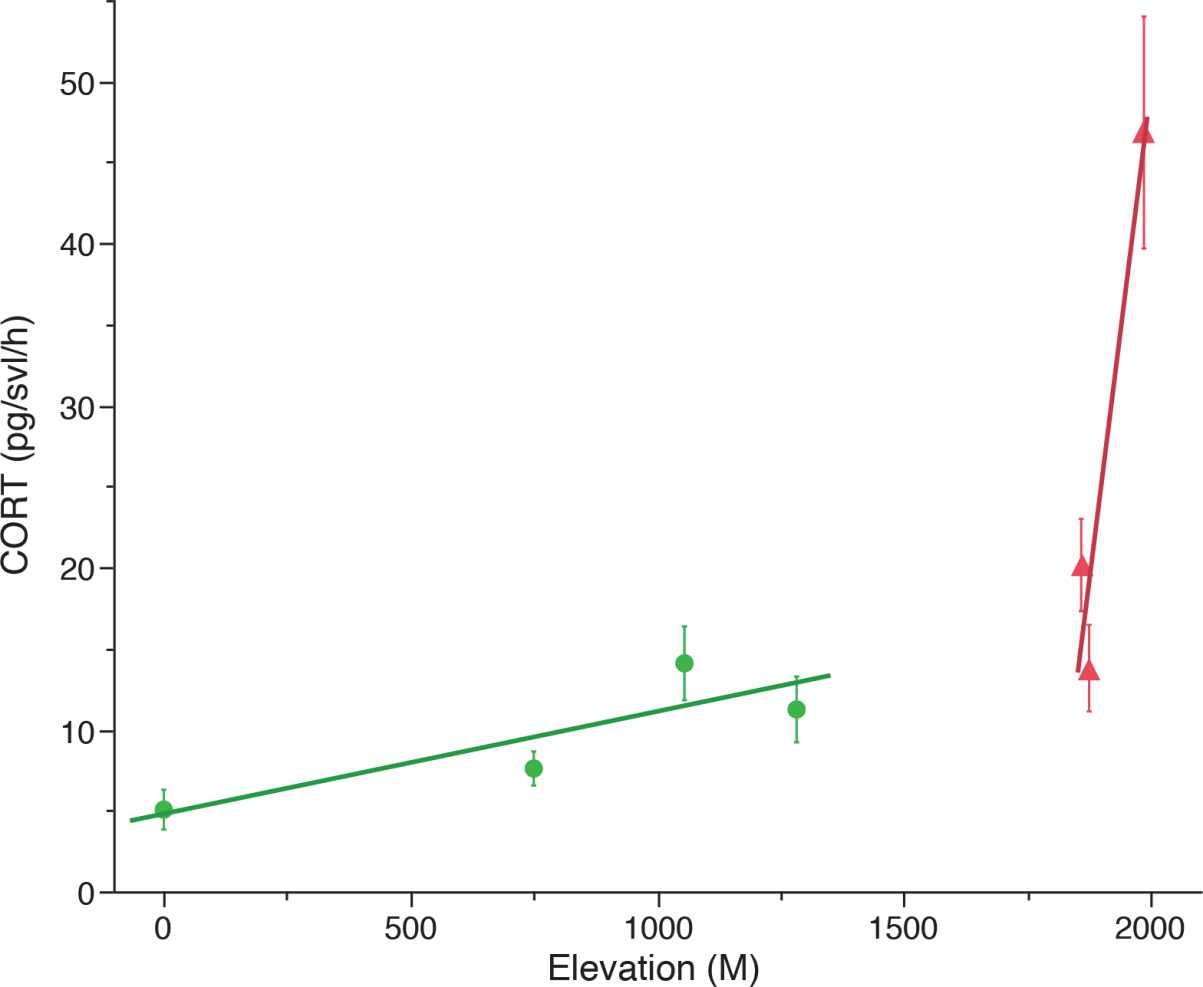
The slope of the relationship between water-borne CORT release rates and elevation across populations of tadpoles. Red indicates infected populations and green indicates uninfected populations of *Alytes obstetricans*.

There was a significant interaction between elevation and infection status (Table 1 and Fig 2). Specifically, CORT release rates increased faster as elevation increased in infected populations when compared against uninfected populations (Fig 3). We measured zero *Bd* load for Iguedri and La Huelga (Picos de Europa National Park), and Toro (Zamora). The *Bd* load for French Pyrenees, Arlet was mean GE = 5.49 ± 1.71 S.E.; range = 25.73–0.00016 GE. There was no relationship between *Bd* load and CORT release rates for the Arlet population (French Pyrenees; GLM: X^2^ = 0.18, *P* = 0.67). Elevation and infection status showed a significant relationship when controlling for CORT (Table 2).

**Table 1:** ANCOVA results for the effects of elevation and *Bd* infection status on water-borne CORT release rates.

Source	Nparm	DF	SS	F Ratio	Prob > F
*Bd* Infection status	1	1	13.71	24.39	<0.0001
Elevation (M)	1	1	9.28	16.51	0.0001
Elevation (M)* *Bd* Infection status	1	1	8.95	15.92	0.0001

**Table 2:** Logistic regression results of the effects of elevation and water-borne Ln CORT release rates on *Bd* infection status.

Source	Nparm	DF	ChiSquare	Prob>ChiSq
Elevation (M)	1	1	119.43	<0.0001
Ln CORT	1	1	2.22e-9	1.00
Ln CORT*Elevation (M)	1	1	6.35e-9	0.99

### Experiment 2: CORT response to *Bd* lineages in *Alytes muletensis*

**Fig 4:**
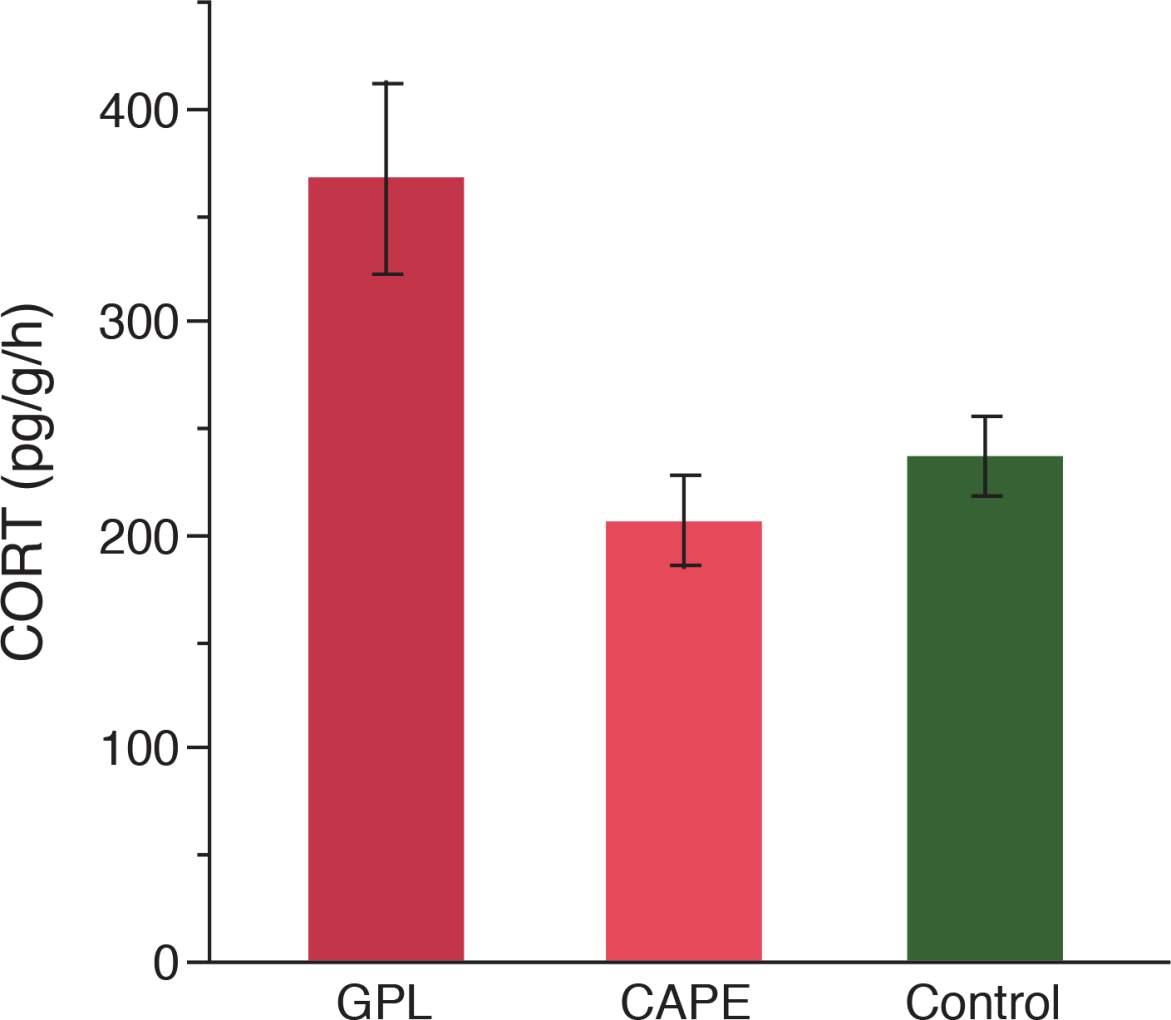
Mean (±SE) water-borne CORT release rates of tadpoles (*Alytes muletensis*) exposed to three lineages of *Bd*.

There was a significant difference in CORT release rates among treatments (ANOVA: F_2,52_ = 8.28, *P* = 0.0008; Fig 4). Based on Tukey’s HSD the tadpoles exposed to *Bd*GPL had significantly higher CORT release rates than *Bd*CAPE (*P* = 0.0005) and the control (*P* = 0.04), but *Bd*CAPE and the control did not significantly differ (*P* = 0.34).

Only one individual exposed to the control had evidence of *Bd* infection in the control at both time points. Three individuals at time 1 (7 days after 4 exposures), and 9 individuals at time 2 (28 days after 4 exposures) were infected with *Bd*CAPE (mean GE value: time 1 = 0.17 ± 0.13 S. E.; time 2 = 12.33 x 5.77 S. E.). Thirteen individuals at time 1 (7 days after 4 exposures) and all from time 2 (28 days after 4 exposures) were infected with *Bd*GPL (mean GE value: time 1 = 12.88 ± 5.56 S. E.; time 2 = 972.89 ± 269.75 S. E.). There was no significant relationship between *Bd* load and CORT release rates in any of the treatments (*Bd*GPL time 1: Kendall’s tau = 0.15, *n* = 17, *P* = 0.40; *Bd*GPL 2: Kendall’s tau = 0.09, *n* = 17, *P* = 0.62; *Bd*CAPE time 1: Kendall’s tau = -0.14, *n* = 19, *P* = 0.47; *Bd*CAPE 2: Kendall’s tau = -0.34, *n* = 19, *P* = 0.06).

### Experiment 3: Righting reflex of metamorphic *Alytes obstetricans*

**Fig 5:**
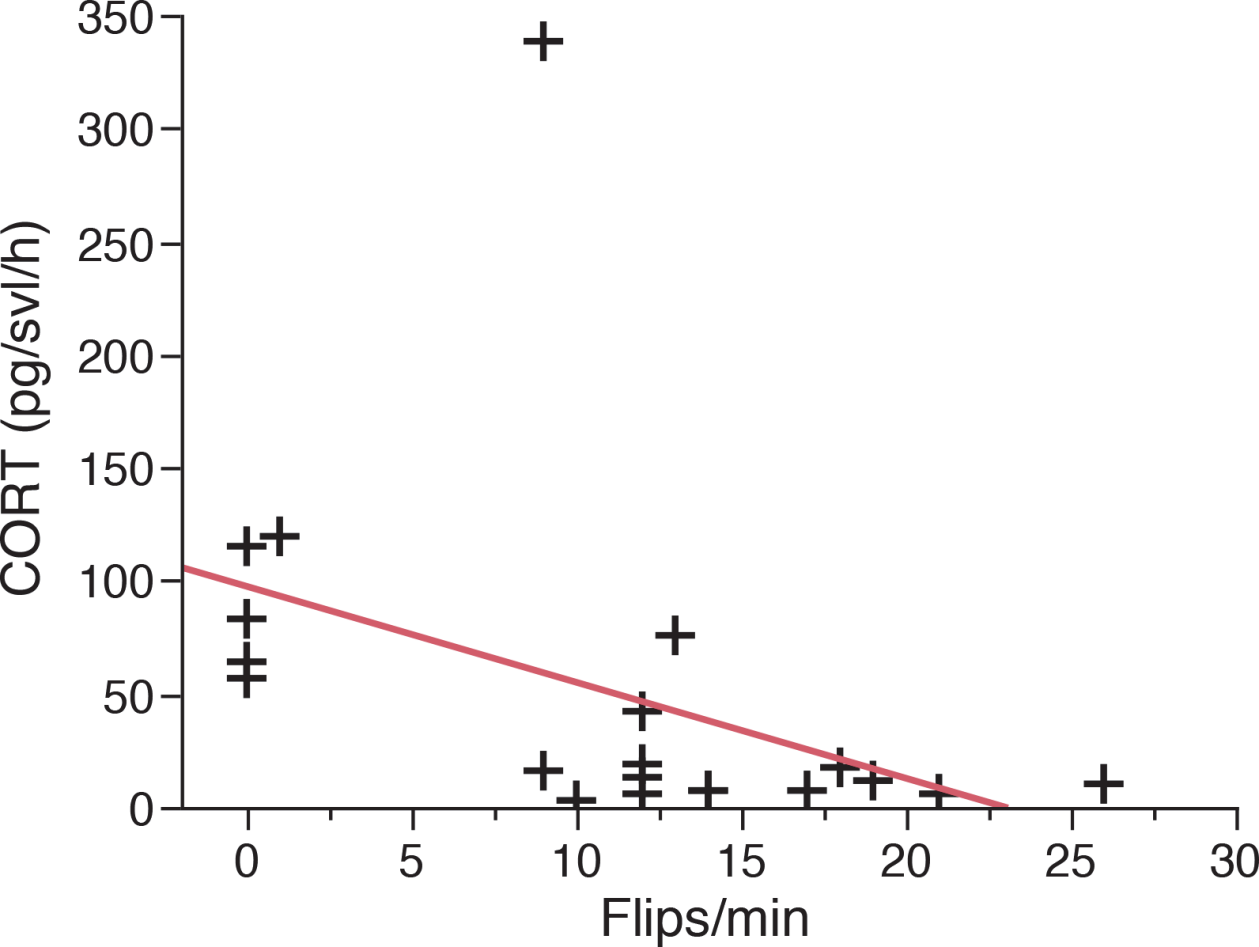
Relationship of water-borne CORT release rates of *Bd* infected metamorphic and the number of flips/min (righting reflex) by *Alytes obstetricians*.

There was a significant negative correlation between CORT release rates and flips/min (Pearson correlation: *n* = 20, *r* = -0.64, *P* = 0.002; Fig 5). There was no significant relationship between CORT release rates and *Bd* load (Kendall’s tau = 0.14, *n* = 20, *P* = 0.40). There was no significant relationship between CORT release rates and *Bd* load (Kendall’s tau = 0.03, *n* = 20, *P* = 0.84).

All Excel files are available from the Texas State University Digital Collections database website https://digital.library.txstate.edu/handle/10877/5462

## Discussion

It is well known that chytridiomycosis has contributed to global amphibian declines; however, some of the mechanisms of action are poorly understood. Here we examined CORT release rates associated with *Bd* infection status in tadpoles. We found that CORT release rates of populations of *Alytes obstetricans* are affected by an interaction between elevation and infection status. Specifically, the slope of the relationship between infection status and elevation differs across infected vs. uninfected populations; in infected populations CORT release rates increase faster as elevation increases than in uninfected populations. This indicates that amphibians found at higher elevations are more stressed, and that infection by *Bd* is associated with additional stress. We also found that tadpoles of *A*. *muletensis* infected with the hypervirulent *Bd*GPL lineage had significantly higher CORT release rates than those infected with the hypovirulent *Bd*CAPE lineage and the control. These results indicate that the aggressiveness of infection by *Bd* drives up CORT levels and that the lineage/virulence is associated with greater stress on the individuals. Additionally, *Bd* infected metamorphs of *A*. *obstetricans* with higher CORT release rates showed a slower righting reflex when turned upside-down than individuals with lower CORT release rates indicating that CORT release rates are associated with a behavior of *A*. *muletensis* that marks active chytridiomycosis and the onset of death.

CORT is also strongly affected by infection as supported by our finding of higher CORT release rates in tadpoles infected by the more virulent lineage of *Bd*. Previously, Gabor et al. [5] found that CORT release rates were significantly higher in a population of *A*. *obstetricans* infected with *Bd*GPL than in a population of *A*. *muletensis* infected with *Bd*CAPE and proposed that the virulence of the lineage drove this difference. Doddington et al. [30] found support for this hypothesis as they showed that significantly more *A*. *muletensis* infected with *Bd*GPL died compared to those infected with *Bd*CAPE. In contrast to our findings, Searle et al. [34] found that tadpoles exposed to elevated CORT and then *Bd* had no effect on *Bd* infection. But, they also found that tadpoles exposed to *Bd* had higher whole-body CORT than unexposed tadpoles. These results suggest that *Bd* is driving CORT levels more so than CORT is driving infection susceptibility.

Higher CORT release rates and greater infection prevalence at higher elevations indicates that CORT release rates are associated with vulnerability. The finding that CORT release rates are greater at higher elevations are consistent with a prior study by Graham et al. [20] but contrasts with the review by Eikenaar et al. [27]. Graham et al. [20] argue that CORT is higher in elevated populations because those populations are ramping up GCs to respond to greater seasonal variability in their environment [35]. Indeed, other studies have found that CORT levels provide an indication of habitat quality [36, 37]. Eikenaar et al. [27] were unclear as to why they found a negative relationship between CORT and elevation in amphibian especially given that they found a positive relationship between CORT and latitude in amphibians. We propose that our link between CORT and elevation is due to temperature variability associated with higher elevations. Further studies with uninfected populations at high elevations would improve our understanding of the relationship between CORT release rates, infection and elevation.

We did not find a relationship between *Bd* load and CORT in any of the experiments. In the field, Gabor et al. [33] found a positive relationship between *Bd* load and CORT for *A*. *muletensis* infected with *Bd*CAPE but they did not find one for *A*. *obstetricans* infected with *Bd*GPL. Peterson et al. [7] also found a positive relationship between *Bd* load and CORT values in the laboratory. They found that CORT significantly increased when *Bd* load was greater than 4000 GE, which was much higher than the *Bd* loads carried by our toads. One hypothesis as to why we did not see a relationship in the field is that environmental factors are also affecting CORT values and infection levels. For example, we found that CORT and elevation are positively correlated and *Bd* infection increased with increasing elevation. Moreover, CORT is negatively correlated with humidity in common toads, *Bufo bufo* [38]. Temperature may also play a factor as Narayan et al. [39] found that CORT increased with temperatures in cane toads. Further, Raffel et al. [40] found that in red-spotted newts, *Notophthalmus viridescens*, immunity decreased with increased temperature variability. Another hypothesis is that the *Bd* diagnostic (e.g., swabbing frogs) is not very accurate in assessing *Bd* loads so it is less likely that we would find a relationship if it existed.

The loss of a righting reflex in metamorphs is consistent with the known effects of infection with *Bd* and occurs at a time when mortality from infection is most commonly found [41, 42]. We found that metamorphs with higher CORT release rates are less able to right themselves after being turned over. In amphibians, there are high energetic costs associated with elevated CORT levels during metamorphosis [43, 44] and this may leave little energy left for other activities and may further account for greater loss of metamorphs either via death/wasting away from infection, or predation, and or the inability to forage or suppression of appetite [7].

In conclusion, our combined field and laboratory approach has supported our hypothesis that *Bd* infection and lineage are strongly associated with the observed higher CORT release rates. Moreover, higher levels of CORT are associated with a loss of righting reflex in metamorphic frogs and are likely associated with the increased mortality of metamorphs found at high elevations [21]. To further disentangle the causal relationship between elevation, CORT and infection will require further experimental manipulation of CORT by increasing and/or blocking CORT production.
